# Determinants of technology-based SMEs’ ability to attract talent with a Master’s degree: Case study of a city in Northeast China

**DOI:** 10.1371/journal.pone.0333184

**Published:** 2025-09-26

**Authors:** Guohong Chen, Lu Xu

**Affiliations:** School of Management, Shenyang Normal University, Shenyang, Liaoning, China; University of Udine, ITALY

## Abstract

Talents with a master’s degree have high-level professional skills and knowledge reserves. They play an important role in overcoming the traditional technological bottlenecks of technology-based small and medium-sized enterprises (SMEs). Taking Shenyang in Northeast China as an example of a talent outflow city, we begin by considering the attraction of technology-based SMEs’ operations to talent with a master’s degree or above. We then use correlation analysis and gray correlation analysis to analyze the influence of technology-based SMEs’ operations on attracting talent with a master’s degree as well as the correlation and correlation degree between the factors. The results show that the number of talents with a master’s degree or above in technology-based SMEs is significantly positively correlated with number of employees (X_9_), total assets (X_3_), total tax paid (X_7_), main business income (X_5_), total indebtedness (X_4_), and total profit (X_6_). These indicators are the determinants of technology-based SMEs’ ability to attract talent with a master’s degree. These results are consistent with the correlation and gray correlation analyses; thus, the results are mutually verified using two different approaches. Recommendations for talent-attraction policies in technology-based SMEs include strengthening the attraction and training of scientific and technological innovation talent, appropriately optimizing assets and liabilities, enhancing core business innovation and development, and promoting steady growth in profit tax payments. Our findings provide a basis for authorities to develop effective strategies and policies to help SMEs attract talent with a master’s degree.

## 1. Introduction

Talent refers to individuals who utilize their unique and superior expertise, knowledge, and leadership skills to contribute to a firm’s performance [1]. Talent, as highly skilled human capital, is an important resource for the development of cities around the world; thus, how to attract, develop, and retain talent is an important issue for contemporary firms [2]. While global cities such as New York, London, and Tokyo are established international gathering places for high-quality talent, cities in developing countries are also striving to create high-quality environments for talent development [3,4]. In 2023, the amount of digital technology talent in China ranked first in the world, accounting for 17% of the global total. However, its high-level digital technology talent only included about 7,000 people, accounting for 9% of the global total. The US, meanwhile, boasts 21,000 high-level digital technology talents, placing China far behind the US [5]. Since China’s “reform and opening up,” the country’s manufacturing industry has developed very rapidly. In terms of total output, China surpassed the US to become the world’s largest manufacturer, with the most stable and complete industrial system [6]. With the rise of new industries and business models for high-end manufacturing, China’s technology talent shortage is as high as five million people, posing serious obstacles to innovation and development [5].

China’s government attaches great importance to the development of small and medium-sized enterprises (SMEs) and has issued many policies to support them. The government’s specific concerns include “supporting the development of small, medium, and micro enterprises,” “supporting the development of enterprises through specialization, refinement, characterization, and novelty,” and “creating a favorable environment for the growth of technology-based small, medium, and micro enterprises.” In 2024, the Third Plenary Session of the Twentieth Central Committee of the Communist Party of China highlighted the need to build a mechanism to promote the development and growth of SMEs through specialization, refinement, characterization, and novelty; encourage technology-based SMEs to increase R&D investment; and increase the proportion of deductions for R&D expenses. Talent, as the main body of technological innovation in technology-based SMEs, can offer rich professional knowledge and skills, innovative thinking, and creativity [7,8]. Developing China’s talent requires accelerating the construction of a national strategic talent force that trains and cultivates first-class scientific and technological leaders and innovation teams while increasing efforts to attract all types of talent.

Talent can bring benefits to the market and society. Excellent talent, measured by the criterion of practical ability, possesses unique professionalism, innovation, and readiness [9]. SMEs can be considered the pillars of a country’s development, making important contributions to both employment and economic development. For an enterprise, excellent talent is a valuable resource that can propel the enterprise forward. The current low profits and low value-added production and operation modes of Chinese SMEs can be attributed to a lack of efficient, high-quality talent, as well as insufficient talent strategies, leading to a status quo for SMEs. Talent holding a master’s degree is a valuable resource with high professional quality and practical experience. Such talent can enhance the technical level and innovation ability of SMEs, promote the progress of technology-based SMEs, and accelerate the industrial development of innovative cities. Attracting and retaining talent with a master’s degree is key to the development and competitiveness of SMEs. It is urgent, then, to build a strategic reserve of talent with a master’s degree for SMEs. Understanding the factors related to attracting talent with a master’s degree can help technology-based SMEs to better identify and attract such talent. Thus, exploring the determinants of SMEs’ ability to attract talent with a master’s degree or above is key to overcoming the technological bottleneck of these SMEs.

This thesis is divided into six chapters, and the organization section is outlined as follows. Chapter 1 introduces the research background and content regarding talent attraction in small and medium-sized enterprises (SMEs). Chapter 2 provides a literature review on talent attraction strategies from macro and micro perspectives, internal and external organizational factors, as well as domestic and international viewpoints. Chapter 3 outlines the main research methods employed to identify the determinants of talent attraction in SMEs. Chapter 4 presents the data analysis results of this study, detailing the decisive factors influencing talent attraction in technology-based SMEs. In Chapter 5, we put forward relevant suggestions based on the data results and the actual development of small and medium-sized technology enterprises. Chapter 6 discusses the limitations of this study and suggests directions for future research.

## 2. Literature review

As the main driving force of economic growth, human capital is a key factor in promoting enterprise development. The accumulation of human capital accelerates the development of science and technology, thereby increasing a country’s total-factor productivity and economic growth [10,11]. Accordingly, various studies have investigated the relationship between talent attraction and enterprise development. Such research has mainly focused on the strategies and effects of talent introduction in specific fields or the effectiveness of organizational talent attraction at the macro level outside the organization and the micro level inside the organization.

Regarding the macro level, studies suggest that talent policies can help organizations attract and retain talent. For example, a research and development support program in France shows that regional subsidies are particularly effective in expanding innovative small and medium-sized enterprises and increasing the number of research and development personnel [8]. As countries develop policies to attract highly skilled researchers, the personal prestige and preferential treatment provided by such policies might play a greater role now than in the past [12]. There are also studies exploring incentive strategies for executives in listed companies, which have found that talent policies have a compensation effect, helping to increase executive compensation and attract talent [13]. The more subsidies the government provides to talents, the more attractive they become [14]. In terms of competitive environments, there was a study used data from listed A-share Chinese technology companies from 2010 to 2019 to investigate the effect of job policy uncertainty in the US on Chinese technology companies. They suggested that when overseas talents return to China, they can mobilize their knowledge base and combine it with high-level skills to reduce the investment costs of Chinese technology manufacturing firms; this increased the willingness of technology-based SMEs to invest and weakens the pricing power of monopoly giants in the market [15]. Furthermore, given the aggregation effect of talent as a scarce resource, the return of overseas talent will continue to attract capital inflow, which will help increase incentives for Chinese technology manufacturing enterprises to compete.

Some studies consider the city as the main factor that affects an organization’s ability to attract excellent talent. For example, examined the factors affecting the retention of excellent scientific and technological talent in China’s developed cities;

Research has found that economic level and living environment are the basic guarantees for attracting and retaining such talents [16]. Meanwhile, the study indicates that the relationship and spatial differences between scientific and technological talents and economic growth in the Yangtze River Delta region from 1998 to 2019, as well as the significant positive impact of economic growth on scientific and technological talents, and vice versa, indicate a long-term equilibrium relationship between scientific and technological talents and economic growth [17]. Analyzing and comparing eight economic regions in China, identified obvious differences between regions. Namely, the eastern and northern coastal regions were in an advantageous position in the competition for talent based on their unique geographic advantages and good economic foundations; their talent attraction was almost 10 times greater than that of the northeastern region [18]. Although career and business remain important components of their lives, the demand for cultural environment is increasing. In addition, the existence of universities plays an important role in their choice of place of residence [19]. A study explored the factors influencing the willingness of talents to settle in Shanghai; They found that housing is the key to accelerating talent’s willingness to settle in cities [20]. China’s Belt and Road Initiative was attractive to Chinese *haigui* (students who return to China after studying abroad) and international talent, but it was not necessarily sufficient for retaining talent in China. Because a lack of local development opportunities often forces talent to leave and seek opportunities elsewhere [21].

The above mentioned studies investigated the factors affecting talent attraction, finding that talent-introduction policies can attract talent [8,12–15]. However, the introduction of policies in various regions has given rise to intense competition for talent [19]. Some studies have analyzed the effects of such factors on talent attraction by taking a city’s economic strength [16–18] and social and cultural environment [19–21] as indicators.

At the micro level, some researchers have examined the effect of talent attraction in enterprises based on specific perspectives. For example, some studies believe that laissez-faire leadership has a positive effect on talent attraction, retention, development, and engagement [22]. When the human resource management (HRM) system is strong, the indirect effect of inclusive leadership on team creativity through team empowerment is stronger, and inclusive leadership plays a key role in attracting talent and fostering team development [23]. The talented managers are a key motivational factor that drives employees to work for a particular enterprise [24]. One study confirmed that creative workspace design has a positive effect on the attractiveness of an organization, and employees are more likely to consider job opportunities in enterprises that offer creative work environments [25]. A creative workspace can be defined as a work environment with unique physical structures and elements that trigger employee creativity and innovation. The work environments of SMEs are poor, and supporting facilities are unable to keep up with changes in the external environment. This leads to team instability and employee dissatisfaction with the environment, thus seriously affecting employees’ work enthusiasm. If enterprises want to be competitive, they need to create a good work environment based on improving their management system and mode of operation [26].

Theories of talent mobility suggest that talented people might leave when working conditions do not meet their expectations; thus, attractive compensation packages can fulfill talented people’s expectations for material rewards. For example, some studies suggested that traditional enterprises should focus on job attributes that align with the values sought by millennials; this could ensure the attractiveness of traditional enterprises in the war for talent [27]. The higher wages can attractive more college graduates [28]. One study was evaluated the US Department of Education’s Science and Mathematics Retention Talent (Smart) Grant Program, found that financial incentives for low-income college students could increase the number of engineering majors [29].

Looking beyond organizational boundaries, there is a study examined the talent acquisition and management strategies of multinational enterprises with various innovation needs, finding that reward and feedback mechanisms can be successful [30]. There was also a study found that SMEs provided a range of intrinsic and extrinsic incentived to retain talent. Such as focusing on an individual’s unique attributes, skills, personality, and character traits [31]. Other studies have identified the industry growth of an enterprise as an important indicator of talent attraction. For example, in a study of Australian SMEs found that corporate branding (e.g., enterprise size, location) and branding processes were key to attracting and retaining employees [32]. The branding is a new indicator of the effect on corporate talent attraction; a study revealed that these enterprises attracted and retained talent mainly through their corporate branding [33]. The corporate social performance can give an enterprise a competitive advantage in the race for talent and positively affect the existing workforce [34].

Talent management is widely recognized as critical for building competitive advantage for enterprises operating in science parks [35,36]. Various studies have concluded that effective talent management strategies are crucial for talent attraction. For example, some scholars suggested that in the implementation process, the HRM system should be optimized in terms of organizational performance, and HRM support for the business should be strengthened [37]. Enterprises must therefore continue to innovate their talent management strategies to meet employee expectations for a good work environment and development opportunities for the mutual benefit of the organization and employees [38]. The talent management directly influenced the affective commitment and turnover intentions of good employees; managers can therefore try to increase affective commitment by matching job tasks with employees’ skills and abilities, thus reducing employees’ willingness to quit [39]. Through human resource management, employees have a stronger sense of belonging, responsibility, competition, and development. Thus, they can contribute to healthy enterprise development, promote harmony among employees, and achieve effective communication and interaction, which will improve team performance [40]. An enterprise’s ability to attract and retain talent depends on its long-term development, which is closely related to the personal interests of employees. The main reasons for talent loss, meanwhile, are hindered development, poor efficiency, rigid mechanisms, and unscientific management. Therefore, to improve conditions for employees, enterprises should seek management mechanisms suitable to their own situation, establish management concepts suitable for long-term development, and improve their economic benefits, market share, and social status. Talent-attraction factors pose a complex, multifaceted issue. The above-mentioned studies found that leadership [22–24], working environment [25,26], welfare benefits [27–30], the development of the enterprise’s industry [31–33], and the talent management system [36–40] were all important factors influencing the mobility and concentration of talent.

Existing research has mainly focused on the external macro-environmental and internal micro-management aspects of enterprises. It has also largely focused on highly marketized and developed urban areas, lacking sufficient attention to brain-drain cities. There is a lack of studies that consider the determinants of technology-based SMEs’ attractiveness to talent with a master’s degree in terms of the scale of operations [31], business conditions [32], and industry development [8,33]. Previous studies suggest that the introduction of highly educated talent can improve firms’ economic strength and increase employment opportunities, thus promoting economic development [8,15,31–33,39].

This study, therefore, aims to grasp the effect of the basic condition, business condition, personnel condition, and scientific and technological innovation of technology-based SMEs on attracting talent with a master’s degree. Shenyang is a heavy industrial and talent outflow city in Northeast China. Taking it as a case study, we consider how an enterprise’s business condition attracts talent with a master’s degree or above. We constructed the index system of the influencing factors of master’s degree or above talent attraction, that includes the three dimensions of scale of operations, business condition, and industry development, as well as 10 secondary indicators. Our research objectives are as follows: (1) Understand the spatial distribution of factors influencing the number of talents with a master’s degree or above in each region of Shenyang through spatial interpolation. (2) Analyze the influence of a firm’s business condition on talent attraction through correlation and association analyses of the factors influencing the number of talents with a master’s degree or above. (3) Finally, formulate effective talent-attraction policy suggestions based on the results.

## 3. Method

### 3.1 Study area and data resources

#### 3.1.1 Study area.

In April 2012, China’s State Council issued the “Opinions of the State Council on Further Supporting the Healthy Development of Small and Micro Enterprises,” proposed to pursue “specialization, refinement, characterization, and novelty,” as well as collaborative and complementary development with large enterprises to accelerate the transformation from factor-driven to innovation-driven development. In July 2013, the Ministry of Industry and Information Technology (MIIT) issued the “Guiding Opinions of MIIT on Promoting the Development of SMEs through Specialization, Refinement, Characterization, and Novelty,” outlining the general idea, key tasks, and measures for promoting SMEs through specialization, refinement, characterization, and novelty. In recent years, Liaoning Province has seen continued increases in the number of technology-based SMEs. There were 7,553 SMEs in 2020. The average annual growth rate of the number of technology-based SMEs and high-tech enterprises in the province over the past three years has been 45.3% and 26.1%, respectively, ranking thirteenth and twelfth nationally. Among them, there are 157 technology-based SMEs in Shenyang and 153 in the nine urban districts. The development of technology-based SMEs in Shenyang still faces many problems. One is a lack of capital investment in science and technology innovation. For technology-based SMEs in Shenyang, the proportion of R&D expenditure in the GDP (2.18%) is lower than the national input intensity (2.44%).

According to the latest data published on the Shenyang Gateway Website (https://www.shenyang.gov.cn/index.html), based on the results of China’s seventh national population census in 2020, the permanent resident population of Shenyang is 9,070,093 people. Compared with the sixth national population census in 2010, which recorded 8,106,171 people, there has been an increase of 963,922 people over the past decade, representing a growth of 11.89%. According to information disclosed by the Human Resources and Social Security Bureau of Shenyang City (https://rsj.shenyang.gov.cn/), in 2024, Shenyang added 1,523 doctoral talents, marking a year-on-year increase of 26.6%. In 2023, Shenyang attracted 22,000 master’s degree holders, marking a year-on-year increase of 69.2%. Although the number of master’s and above talents has significantly increased in the past two years, compared to rapidly developing cities of similar type, Shenyang still faces a substantial gap in the number of such talents, which is insufficient to support technological innovation in small and medium-sized technology-based and the city’s overall innovative development. Further, there is a lack of scientific and technological innovation talent, a large outflow of excellent talent, a wait-and-see attitude among talent, and no talent-attraction or incentive mechanisms. Large gaps remain in the supply of talent in certain key industries and fields in Shenyang. There is a lack of composite leadership talent with strong scientific and technological literacy and enterprise management ability, and there is still a shortage of R&D talent who can master the concepts and methods of advanced product R&D. Shenyang can therefore be considered a representative area for research on technology-based SMEs’ ability to attract talent. This study conducted field research on more than 30 enterprises and actively communicated with the Shenyang City Government. By adopting a full-caliber survey, it obtained all the data of 153 small and medium-sized technology-based enterprises in Shenyang that met the requirements. Through grey correlation analysis, this study analyzed the correlation degree of each index with the attraction of master’s degree or above talents in small and medium-sized technology-based enterprises in Shenyang.

#### 3.1.2 Data resources.

In order to identify the determinants of technology-based SMEs’ talent attraction and make targeted suggestions, we interviewed six enterprise chambers of commerce (associations), visited more than 30 enterprises, and surveyed the development situations of 153 technology-based SMEs in Shenyang. We use scale of operations, business condition, and industry development to explore the effect on the number of talents with a master’s degree or above. Specific indicators include whether it is an above-scale enterprise, enterprise scale, total assets, total indebtedness, main business income, total profit, total tax paid, industry type, number of employees, and R&D investment as a proportion of operating income.

### 3.2 Methods

First, by reading relevant research, we focused on the latest policies regarding the development of small and medium-sized technology-based enterprises in our country and the Shenyang City Government’s macro plan for the development of small and medium-sized technology-based enterprises. This led us to summarize three dimensions: business scale, operational status, and industry development, along with ten secondary indicators, as detailed in Table 1. Second, the data for this study was primarily obtained through two methods: some data was collected from actual field visits and surveys, while other data were gathered through actively communicating with the Shenyang Municipal Industry and Information Technology Bureau, and distributing questionnaires through the Bureau. Finally, the primary data obtained from these two sources were organized to form the database for this study. Third, before conducting Grey correlation analysis, SPSS software was used to test the data to determine if it followed a normal distribution. Lastly, we use correlation models to analyze the relationship between the distribution of the number of talents with a master’s degree or above and the 10 above mentioned factors. We use gray correlation analysis to evaluate the correlations among the 10 indicators based on the principle of maximum correlation to identify the determinants of technology-based SMEs’ attractiveness to talent with a master’s degree.

**Table 1:** The index system that affects the number of talents with master’s degree or above in small and medium-sized science and technology enterprises.

Dimensions	Factors
Scale of operations	Whether above-scale enterprises (*X*_1_)
	Enterprise scale (*X*_2_)
	Total assets (*X*_3_)
	Total indebtedness (*X*_4_)
Business conditions	Main business incomes (*X*_5_)
	Total profits (*X*_6_)
	Total tax paid (*X*_7_)
Industry development	Industry types (*X*_8_)
	Number of employees (*X*_9_)
	R & D investment as a proportion of operating incomes (*X*_10_)

#### 3.2.1 Correlation analysis model.

Correlation analysis is used to study the relationship between quantitative data, including whether there is a relationship and the degree of closeness of the relationship. In this study, we mainly use it to study the relationship between the variable *Y*—number of talents with a master’s degree or above—and the 10 correlation indicators: whether it is an above-scale enterprise (*X*_1_), enterprise scale (*X*_2_), total assets (*X*_3_), total indebtedness (*X*_4_), main business income (*X*_5_), total profit (*X*_6_), total tax paid (*X*_7_), industry type (*X*_8_), number of employees (*X*_9_), and R&D investment as a proportion of operating income (*X*_10_) (Equation (2)):


$$\begin{array}{c} p_{x,y=\frac{E(X,Y)-E(X)E(Y)}{\sqrt{E(X{)}^{\text{2}}-(E(X))\text{2}}-\sqrt{E(Y{)}^{\text{2}}-(E(Y))\text{2}}}} \end{array}$$ (1)

A larger absolute value of the correlation coefficient suggests a stronger correlation: The closer the correlation coefficient is to 1 or −1, the stronger the correlation, and the closer the correlation coefficient is to 0, the weaker the correlation.

#### 3.2.2 Grey correlation analysis.

We use gray correlation analysis to evaluate the degree of development of technology-based SMEs. Gray correlation analysis determines whether the parent series *Y* and a number of characteristic series *X* are closely linked by determining the degree of geometric similarity between them, which reflects the degree of correlation between the curves. We use this method to evaluate the correlation between the number of talents with a master’s degree *Y* and our 10 indicators. Generally, the discriminatory effect is usually better when *ρ = 0*.5, and the degree of correlation is obtained in accordance with the magnitude of the correlation (Equation (3)):


$$\delta_{i}(\text{k})=\frac{\begin{array}{c} min\;\\ i \end{array}\begin{array}{c} min\\ k \end{array}\left|Y(k)-X_{i}(k)\right|+\rho \begin{array}{c} max\;\\ i \end{array}\begin{array}{c} max\\ k \end{array}|Y(k)-X_{i}(k)|}{\left|Y(k)-X_{i}(k)\right|}$$ (2)

where $\delta_{i}\;$ is the gray correlation coefficient; *ρ* is the discrimination coefficient, taking a value of 0.5; *i* is the *ith* factor; and *k* is the *kth* level number. According to the principle of maximum correlation, the factor corresponding to the maximum value of correlation has the greatest influence on the development of the enterprise; as the correlation decreases, the corresponding factor has less influence.

## 4. Results

### 4.1 Correlation between the number of introduced talents and enterprise development

**Fig 1:**
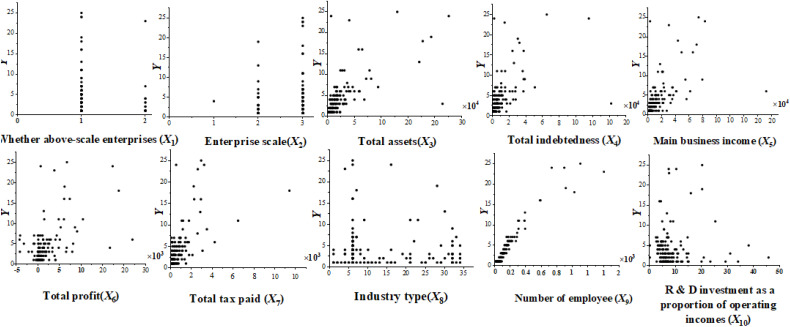
Scatter plot of the distribution of postgraduate talents with various influencing factors.

Before the correlation analysis, we need to test the data to determine whether it meets normal distribution. It is convenient to test the normality of the correlation indicators by making a scatterplot of the correlation indicator data. A normal distribution curve is a smooth curve with two low sides and a high center, and the scatterplot can show the distribution of the 10 indicators. Whether it is an above-scale enterprise (*X*_1_) and enterprise scale (*X*_2_) are linearly distributed and do not satisfy the normal distribution curve law. Total assets (*X*_3_), total indebtedness (*X*_4_), main business income (*X*_5_), total profit (*X*_6_), total tax paid (*X*_7_), and number of employees (*X*_9_) are mainly gathered in the lower left corner, presenting a lumpy shape, thus not satisfying normal curve distribution. R&D investment as a proportion of operating income (*X*_10_) overall shows a primary function of the distribution and does not satisfy the normal distribution curve law. Industry type (*X*_8_) has a semicircular distribution around the coordinate axis in the first quadrant and does not satisfy smooth curve distribution (Fig. 1). Therefore, none of the 10 factors satisfy normal distribution.

Since the number of talents with a master’s degree or above and the 10 factors do not satisfy normal distribution, we use Pearson’s correlation coefficient to indicate the strength of the correlation. Using SPSS software to analyze the data, the results are as p-values in Table 2, which indicate the probability of sample data occurring under the null hypothesis. If p-values less than 0.05 or 0.01, the significance level is reached. The p-values for the number of postgraduate talents and Total assets (*X*_3_), Total indebtedness (*X*_4_), Main business incomes (*X*_5_), Total profits (*X*_6_), Total tax paid (*X*_7_), and Number of employees (*X*_9_) are all less than 0.01, indicating that these six influencing factors have a significant correlation with the number of postgraduate talents. The correlation coefficient r represents the relationship between two variables; the larger the absolute value of r, the stronger the correlation between the two variables. In Table 2, we use Pearson’s correlation coefficient to indicate the strength of the correlation (Table 1). The correlation coefficients between the number of talents with a master’s degree or above and number of employees (*X*_9_), total assets (*X*_3_), total tax paid (*X*_7_), main business income (*X*_5_), total indebtedness (*X*_4_), and total profit (*X*_6_) are significant at the 0.01 level. Their correlation coefficients are 0.949, 0.666, 0.607, 0.551, 0.521, and 0.498, respectively, indicating that there is a significant positive correlation between the number of talents with a master’s degree or above and these six factors. The correlation coefficients between the number of talents with a master’s degree or above and R&D investment as a proportion of operating income (*X*_10_) and enterprise scale (*X*_2_) are 0.054 and 0.050, respectively, indicating that there is a weak positive correlation between the number of talents with a master’s degree or above and these two factors. The correlation coefficients between the number of talents with a master’s degree or above and whether it is an above-scale enterprise (*X*_1_) and industry type (*X*_8_) are −0.109 and −0.079, respectively, indicating that the number of talents with a master’s degree or above has a weak negative correlation with these two factors. The above results show that the number of talents with a master’s degree or above has an important influence on scale of operations, business condition, and industry development. Therefore, the introduction of talent can be regarded as an effective strategic means to enhance the overall strength and competitiveness of an enterprise.

**Table 2:** Analysis results between postgraduate talents and influencing factors.

Dimensions	Factors	r	P	Samples	Average value	Standard deviation	Variance
Scale of operations	Whether above-scale enterprises (*X*_1_)	−0.109	0.18	153	1.144	0.352	0.124
	Enterprise scale (*X*_2_)	0.050	0.537	153	2.791	0.424	0.180
	Total assets (*X*_3_)	0.666**	0	153	23808.618	37433.022	1401231114.965
	Total indebtedness (*X*_4_)	0.521**	0	153	9760.971	17304.930	299460589.433
Business conditions	Main business incomes (*X*_5_)	0.551**	0	153	14845.505	20756.209	430820210.588
	Total profits (*X*_6_)	0.498**	0	153	1761.808	3669.338	13464038.330
	Total tax paid (*X*_7_)	0.607**	0	153	746.882	1266.081	1602961.065
Industry development	Industry types (*X*_8_)	−0.079	0.331	153	155.373	201.689	40678.485
	Number of employees (*X*_9_)	0.949**	0	153	8.401	6.568	43.144
	R & D investment as a proportion of operating incomes (*X*_10_)	0.054	0.505	153	13.013	10.500	110.250

Note: *p < 0.05, **p < 0.01

### 4.2 Evaluation based on gray correlation

In Table 3, number of employees (*X*_9_) has the largest correlation with the number of talents with a master’s degree or above *Y*, indicating that talent clustering is still the most important for attracting talent with a master’s degree or above. Total assets (*X*_3_), main business income (*X*_5_), total indebtedness (*X*_4_), total tax paid (*X*_7_), and total profit (*X*_6_) are more strongly related to the number of talents with a master’s degree or above *Y*. This is more consistent with the correlation analysis results, thus confirming that the introduction of highly educated talent is more closely related to the scale of operations and business condition. In addition, the introduction of highly educated talent might help improve the economic strength of an enterprise and increase employment opportunities, thus promoting economic development. The correlation between industry type (*X*_8_), whether it is an above-scale enterprise (*X*_1_), enterprise scale (*X*_2_), and R&D investment as a proportion of operating income (*X*_10_) and the number of talents with a master’s degree or above is not sufficiently significant. This indicates that in reality, these factors are not determinants of the number of introduced talents *Y*. This result is basically consistent with the correlation analysis results and can be mutually verified.

**Table 3:** Correlation results between the number of postgraduate talents and influencing factors.

Dimensions	Factors	Correlation	Rank
Scale of operations	Whether above-scale enterprises (*X*_1_)	0.845	7
	Enterprise scale (*X*_2_)	0.800	10
	Total assets (*X*_3_)	0.919	2
	Total indebtedness (*X*_4_)	0.909	4
Business conditions	Main business incomes (*X*_5_)	0.910	3
	Total profits (*X*_6_)	0.890	6
	Total tax paid (*X*_7_)	0.907	5
Industry development	Industry types (*X*_8_)	0.809	9
	Number of employees (*X*_9_)	0.958	1
	R & D investment as a proportion of operating incomes (*X*_10_)	0.845	8

In conclusion, there is a significant positive correlation between the number of talents with a master’s degree or above and the six factors of number of employees (*X*_9_), total assets (*X*_3_), total tax paid (*X*_7_), main business income (*X*_5_), total indebtedness (*X*_4_), and total profit (*X*_6_). These six factors are the determinants of talent attraction for technology-based SMEs.

## 5. Discussion and policy proposals

### 5.1 Policy proposal

This study is to break through the limitations of enterprises as passive recipients of policies, and the government, as a policy maker and implementer, can guide the benign development of enterprises. Therefore, based on the influencing factor of Number of employee (X9), we put forward the policy suggestions to strengthen the attraction and cultivation of scientific and technological innovation talents. Combined with Total assets (X3) and Total indebtedness (X4), we put forward the policy suggestions to optimize the reasonable scale of assets and liabilities. Based on Main business income (X5), it puts forward policy suggestions to strengthen the innovation and development of core business. Combined with Total profit (X6) and Total tax paid (X7), it puts forward policy suggestions to promote the steady growth of profit and tax payment.

#### 5.1.1 Enhancing the cultivation and attraction of technological innovation talents.

Encourage small and medium-sized technology enterprises to recruit high-level talents by implementing favorable talent policies, thereby building research capabilities for technological innovation. Support technological SMEs in intensifying efforts for flexible talent deployment, exploring the implementation of awards and incentives for high-level technological talents, and guiding them to establish technology-oriented scholar workstations to nurture leading talents in science and technology. In response to the national requirements of developing new quality productive forces, leverage the educational strengths of Liaoning Province, which boasts 114 universities and 6 research institutes affiliated with the Chinese Academy of Sciences, to establish a comprehensive personnel training system. Encourage universities and vocational colleges to tailor courses to the talent needs of SMEs, providing training for innovative talents. Guide and support high-level talents from universities and research institutions to take up positions or engage in part-time roles in technological SMEs.

#### 5.1.2 Optimizing the right size of assets and liabilities.

Regularly map and investigate small and medium-sized science and technology enterprises in Shenyang, do a good job of combing basic data, take the scale of assets and liabilities as one of the important evaluation indicators, and select high-quality small and medium-sized science and technology enterprises in different industries. According to the gradient cultivation system of small and medium-sized science and technology enterprises and high-tech enterprises, we will increase the cultivation and support for small and medium-sized science and technology enterprises, do a good job in guiding and serving enterprises, strengthen the whole chain cultivation of small and medium-sized science and technology enterprises, and continue to grow the main body of innovation, and continuously develop the new kinetic energy.

#### 5.1.3 Strengthening core business innovation and development.

Guiding science and technology-based small and medium-sized enterprises to benchmark against provincial innovation-oriented enterprises and high-tech enterprises, and stimulating the innovation vitality of enterprises. Improve the mechanism of science and technology innovation of small and medium-sized enterprises, support enterprises to carry out core technology research and development through key areas of research and development programs, set up technology research and development topics for science and technology-based small and medium-sized enterprises, and encourage small and medium-sized science and technology-based enterprises to participate in the formation of leading enterprises to form innovation consortia and jointly undertake scientific research projects. Improve the integration mechanism, give full play to the role of science and technology demonstration zones such as Hunnan District, Shenyang City, as a demonstration base for dual innovation, and support large-scale enterprises, especially “chain master” enterprises, to integrate science and technology-based small and medium-sized enterprises into the industrial chain and supply chain system through industrial ties, upstream and downstream support, division of labor and technology diffusion, etc., so as to promote the integration and development of small and medium-sized enterprises.

#### 5.1.4 Promoting steady growth in profit tax payments.

To formulate full-cycle, multi-angle and multi-level financial and tax support policies for small and medium-sized science and technology enterprises, ensure that the policies are put in place, and stimulate enterprises’ enthusiasm and motivation for innovation. Strengthen the synergy of financial and fiscal policies, and cooperate with the provincial People’s Bank of China and the provincial financial authorities to help banks launch targeted science and technology loans, credit loans, patent loans and other types of science and technology loans. Relying on the Northeast Science and Technology Market and other innovation platform carriers to create a platform for science and technology investment and financing, the introduction of domestic and foreign heavyweight angel funds, venture capital and other science and technology financial institutions to serve small and medium-sized science and technology-based enterprises. Implement tax and fee reduction policies, formulate a list of tax and fee incentives, accurately push tax policies, reduce the production and operation costs of small and medium-sized science and technology enterprises, and optimize tax services and management.

### 5.2 Discussion

Human activity affects sustainable development. In less developed regions, as human activity increases, so does the interaction between humans and local production and ecological space [41]. In China, talent mainly flows out from Central China, Northwest China, and Northeast China. Jilin and Liaoning Provinces have become the typical regions in China with high brain drain, and the phenomena of the Midwest crisis and the Northeast dilemma still exist. Northeast China, which is an important industrial and agricultural base, is strategically important for maintaining China’s food, ecological, energy, and industrial security, making it crucial for overall national development. Therefore, we offer a macro-level policy analysis of the factors affecting talent attraction in the Northeast based on a micro-level analysis of SMEs in a typical brain-drain city in China. Our results confirm that the business conditions of enterprises are important determinants that affect the ability of organizations to attract talent with a master’s degree. Finally, we propose that the educational advantages of Liaoning Province should be utilized to increase talent attraction in Northeast China. We also make policy suggestions regarding how to strengthen the attraction and cultivation of talent with a master’s degree or above in brain-drain cities. For example, we should strengthen the attraction and cultivation of scientific and technological innovation talent and promote the cultivation of scientific and technology-based SMEs. We should also improve SMEs’ scientific and technological innovation capabilities, improve the policy system that benefits enterprises at multiple levels, deepen industry–university–research institute cooperation using platforms, and formulate talent-attraction policies in accordance with local characteristics.

In recent years, the trend of labor outflow from Northeast China has become increasingly obvious, which is an important factor restricting the region’s economic development and talent attraction. Talent is a crucial resource, and technology-based SMEs’ innovation, as a knowledge-intensive activity, is important for promoting the development of cities and SMEs alike [42,43]. As pillars of national economic development, technology-based SMEs help improve a country’s employment and economic level. If they are to develop, SMEs need to attract and retain talent [44]. However, many internal and external organizational factors affect high-quality talent acquisition. Analyzing the typical brain-drain city of Shenyang, we find that talent attraction is mainly based on the size of the enterprise and its economic output. This contrasts with previous studies of talent attraction in developed cities, which focused on the knowledge culture and urban inclusiveness [16–21]. Other studies indicate that branding is a new indicator that affects firms’ talent attraction, and corporate social performance can give enterprises a competitive advantage in the competition for talent [30,31]. Shares are an important part of the company’s capital structure, reflecting the capital strength. Equity financing can expand research and development and market, and ESOP can motivate employees to improve the company’s innovation ability. Some studies include executive equity incentive into the theoretical analysis framework of enterprise environmental innovation, and the regression analysis model shows that executive equity incentive plays an important role in actively regulating green innovation. Therefore, the capital strength of enterprises and the attraction of enterprise talents form a virtuous circle. This research and our study have the same analysis of the impact of enterprise capital strength on the construction of enterprise talent team. The difference is that the study discusses the impact of equity incentives on corporate executives from an equity perspective. According to this study, executive stock incentive is an important tool to attract talents and achieve long-term development goals, and has a profound impact on the development of green innovation of enterprises [45]. There are also studies similar to our research method, using GRA to build the human resource crisis early warning index system of bearing enterprises. Finally, it is found that the five indicators that have the biggest impact on the human resource crisis of enterprises are: employee turnover rate, internal personnel turnover rate, personnel shortage rate, talent introduction rate and guidance failure rate. The study concluded that the introduction and loss of talents have a significant impact on the stability, production efficiency, economic benefit and competitiveness of bearing enterprises. The conclusions of this study also provide support for the findings of this paper [46]. This is consistent with our results, which also indicate that a firm’s business condition is an important factor that affects talent attraction.

This study has some limitations. First, the research period is 2020, which is when the COVID-19 pandemic had enormous effects on the global society and economy. Talent shortages [47] led to the closure of businesses and the disruption of the global industrial chain. Second, by focusing on talent with a master’s degree or above, we lack an in-depth exploration of other types of talent; thus, our findings might be limited to certain specificities. Future research can aim to further deepen our understanding of talent-attraction mechanisms and explore more effective talent-attraction strategies and practical experiences. Moreover, attention should be paid to the differences in the talent-attraction strategies of enterprises in different industries and of different sizes. Research should also focus on the trends and challenges of talent mobility in the context of globalization, which could make the results more generalizable.

## 6. Conclusion

This study conducts a case study of Shenyang, a city in Northeast China. Focusing on firms’ ability to attract talent with a master’s degree or above, we explore the determinants of talent attraction and make suggestions for attracting talent in technology-based SMEs. The conclusions are as follows:

(1) There is a significant positive relationship between the number of talents with a master’s degree or above and six factors: number of employees (*X*_9_), total assets (*X*_3_), total tax paid (*X*_7_), main business income (*X*_5_), total indebtedness *X*_4_), and total profit (*X*_6_). These factors are the determinants of talent attraction for technology-based SMEs.
(2) Talent attraction policies for technology-based SMEs should be formulated by considering the key factors of talent attraction in four aspects: strengthening the attraction and cultivation of scientific and technological innovation talent, optimizing the scale of assets and liabilities, strengthening the innovation and development of core businesses, and promoting the steady growth of profit tax payments.

## Supporting information

S1 Appendix The initial data of technology-Based SMEs’ Ability to Attract Talent with a Master’s Degree. (DOCX)
