# Supplementary material for: Determinants of technology-based SMEs’ ability to attract talent with a Master’s degree: Case study of a city in Northeast China
Source: PLoS One. 2025 Sep 26;20(9):e0333184. doi: 10.1371/journal.pone.0333184 (PMC12468759; doi:10.1371/journal.pone.0333184)
Supplement: S1 Appendix — (DOCX) [file pone.0333184.s001.docx]

**S1 Appendix**  The initial data of technology-Based SMEs’ Ability to Attract Talent with a Master’s Degree

| Num. | District Number | Number of master talents introduced | Whether above-scale enterprises (Yes = 1, no = 2) | Enterprise scale (Middle = 2, small = 3) | Total assets (Ten thousand yuan) | Total indebtedness (Ten thousand yuan) | Main business income (Ten thousand yuan) | Total profit (Ten thousand yuan) | Total tax paid (Ten thousand yuan) | Industry type (Ten thousand yuan) | Number of employee (Ten thousand yuan) | R & D investment as a proportion of operating income (%) |
| --- | --- | --- | --- | --- | --- | --- | --- | --- | --- | --- | --- | --- |
| 1 | 1 | 1 | 1 | 2 | 6588 | 2377 | 3062 | 2 | 172 | 29 | 58 | 2.74 |
| 2 | 1 | 2 | 1 | 2 | 5394 | 1777 | 4073 | 760 | 404 | 34 | 109 | 0.1 |
| 3 | 1 | 1 | 1 | 3 | 5171 | 2912 | 5148 | 183 | 175 | 9 | 60 | 5.4 |
| 4 | 1 | 5 | 1 | 2 | 17402 | 8189 | 10856 | -961 | 569 | 32 | 160 | 6 |
| 5 | 1 | 25 | 1 | 3 | 129471 | 64923 | 73776 | 6734 | 2930 | 6 | 1100 | 20.2 |
| 6 | 1 | 1 | 1 | 3 | 17377 | 8371 | 5186 | -969 | 59 | 21 | 56 | 9.04 |
| 7 | 1 | 3 | 1 | 3 | 14570 | 5293 | 8925 | 531 | 465 | 4 | 143 | 10.72 |
| 8 | 1 | 7 | 2 | 3 | 38126 | 27638 | 36037 | 4373 | 945 | 6 | 170 | 3 |
| 9 | 2 | 2 | 1 | 2 | 16262 | 11283 | 4961 | 311 | 73 | 27 | 76 | 6.33 |
| 10 | 2 | 1 | 2 | 3 | 1681 | 876 | 2000 | 242 | 57 | 6 | 25 | 12.3 |
| 11 | 2 | 6 | 1 | 3 | 34775 | 20550 | 3688 | -4211 | 179 | 22 | 272 | 5.12 |
| 12 | 2 | 2 | 1 | 3 | 16103 | 8295 | 9118 | 1361 | 635 | 26 | 77 | 9.28 |
| 13 | 2 | 1 | 2 | 3 | 2033 | 262 | 1786 | 213 | 125 | 5 | 43 | 6 |
| 14 | 2 | 5 | 1 | 3 | 17545 | 10088 | 12420 | 1564 | 743 | 6 | 176 | 0.1 |
| 15 | 3 | 11 | 1 | 3 | 27051 | 10642 | 20914 | 6056 | 1177 | 23 | 311 | 8 |
| 16 | 3 | 7 | 1 | 3 | 93888 | 50600 | 23162 | 699 | 905 | 6 | 230 | 2.46 |
| 17 | 3 | 4 | 1 | 3 | 15676 | 5719 | 12774 | 16712 | 401 | 6 | 123 | 10.33 |
| 18 | 3 | 2 | 1 | 3 | 2841 | 1385 | 2498 | 287 | 135 | 13 | 98 | 5.6 |
| 19 | 3 | 3 | 1 | 3 | 9184 | 6279 | 9806 | 314 | 342 | 6 | 88 | 7.36 |
| 20 | 4 | 2 | 1 | 3 | 13831.59 | 7621.60 | 8049.30 | 490.67 | 207 | 6 | 56 | 4.25 |
| 21 | 4 | 1 | 2 | 3 | 2729 | 1670 | 1782 | -24 | 42 | 6 | 11 | 5.33 |
| 22 | 4 | 6 | 1 | 3 | 49570 | 27171 | 172169 | 1259 | 1583 | 6 | 211 | 2.61 |
| 23 | 4 | 5 | 1 | 3 | 29147 | 10731 | 30685 | 8178 | 1357 | 6 | 162 | 3.5 |
| 24 | 4 | 4 | 1 | 3 | 8100 | 1240 | 7057 | 210 | 128 | 6 | 120 | 5.3 |
| 25 | 4 | 5 | 1 | 3 | 15358 | 10560 | 10534 | 2407 | 1158 | 6 | 147 | 12.04 |
| 26 | 4 | 6 | 1 | 3 | 58138 | 36894 | 40975 | 3012 | 1370 | 6 | 186 | 4.55 |
| 27 | 4 | 24 | 1 | 3 | 225341 | 115127 | 82949 | 17344 | 3210 | 6 | 894 | 7.35 |
| 28 | 4 | 1 | 2 | 3 | 2258 | 1382 | 3223 | 76 | 64 | 16 | 56 | 5.18 |
| 29 | 4 | 1 | 2 | 3 | 8594 | 3949 | 3520 | 184 | 122 | 5 | 54 | 5 |
| 30 | 4 | 6 | 1 | 3 | 59288 | 23887 | 24469 | 113 | 1734 | 6 | 208 | 8.34 |
| 31 | 4 | 3 | 1 | 3 | 28238 | 5159 | 14929 | 1149 | 918 | 6 | 107 | 11 |
| 32 | 4 | 2 | 1 | 3 | 7372 | 5058 | 6913 | 398 | 255 | 6 | 72 | 14.38 |
| 33 | 4 | 3 | 1 | 3 | 5452 | 2550 | 4777 | 234 | 51 | 6 | 101 | 5.9 |
| 34 | 4 | 6 | 1 | 3 | 42004 | 10767 | 41479 | 21906 | 4230 | 6 | 223 | 3.19 |
| 35 | 4 | 3 | 1 | 3 | 34548 | 10991 | 21336 | 7998 | 2086 | 6 | 114 | 3 |
| 36 | 4 | 4 | 1 | 3 | 32792 | 15144 | 10843 | 4840 | 1082 | 6 | 112 | 5.31 |
| 37 | 4 | 2 | 1 | 3 | 7908 | 3367 | 6526 | -113 | 232 | 6 | 76 | 7.74 |
| 38 | 4 | 2 | 1 | 3 | 9314 | 2192 | 3607 | 730 | 182 | 6 | 65 | 45.71 |
| 39 | 4 | 24 | 1 | 3 | 6242 | 2002 | 3127 | 663 | 530 | 16 | 732 | 10.27 |
| 40 | 4 | 1 | 1 | 3 | 2234 | 337 | 2578 | 396 | 226 | 16 | 36 | 12.06 |
| 41 | 4 | 7 | 1 | 3 | 12531 | 4359 | 7548 | 31 | 317 | 6 | 175 | 7.25 |
| 42 | 4 | 4 | 1 | 3 | 49724 | 16413 | 23947 | 6698 | 3056 | 6 | 132 | 3.04 |
| 43 | 4 | 19 | 1 | 2 | 192782 | 30157 | 43616 | 6214 | 2213 | 28 | 913 | 20.22 |
| 44 | 4 | 1 | 1 | 3 | 2008 | 1167 | 2121 | -37 | 61 | 25 | 25 | 6.35 |
| 45 | 4 | 1 | 1 | 3 | 8216 | 2219 | 2396 | 175 | 81 | 6 | 58 | 29.2 |
| 46 | 4 | 3 | 1 | 3 | 6689 | 3356 | 3577 | 146 | 181 | 26 | 103 | 8.6 |
| 47 | 4 | 5 | 1 | 2 | 12425 | 6477 | 32418 | -3222 | 324 | 29 | 169 | 4 |
| 48 | 4 | 1 | 1 | 3 | 4329 | 1759 | 2770 | 170 | 43 | 16 | 48 | 4.28 |
| 49 | 4 | 1 | 2 | 3 | 3270 | 346 | 1344 | 740 | 142 | 2 | 25 | 34 |
| 50 | 4 | 4 | 1 | 3 | 17354 | 4786 | 5163 | 186 | 57 | 6 | 123 | 13 |
| 51 | 4 | 2 | 1 | 3 | 7089 | 3321 | 5216 | 251 | 317 | 6 | 93 | 4.67 |
| 52 | 5 | 2 | 1 | 3 | 10305 | 6913 | 4137 | 465 | 257 | 6 | 60 | 6 |
| 53 | 5 | 2 | 1 | 3 | 4941 | 1309 | 6623 | 968 | 375 | 20 | 75 | 10.64 |
| 54 | 5 | 1 | 1 | 3 | 6216 | 79 | 4687 | 375 | 212 | 25 | 46 | 6.6 |
| 55 | 5 | 1 | 1 | 2 | 4901 | 3122 | 7361 | 278 | 150 | 1 | 86 | 6 |
| 56 | 5 | 1 | 1 | 2 | 11592 | 7398 | 15879 | 165 | 210 | 28 | 46 | 7.46 |
| 57 | 5 | 5 | 1 | 3 | 16695 | 2666 | 14755 | 1769 | 1110 | 6 | 171 | 4.7 |
| 58 | 5 | 4 | 1 | 3 | 12552 | 5504 | 21093 | 3403 | 561 | 7 | 119 | 4.03 |
| 59 | 5 | 3 | 1 | 3 | 14203 | 8918 | 6894 | -1339 | 246 | 6 | 122 | 8.9 |
| 60 | 5 | 3 | 2 | 3 | 31114 | 21200 | 16441 | 1020 | 275 | 6 | 98 | 9.1 |
| 61 | 5 | 7 | 1 | 3 | 46365 | 10473 | 35232 | 1104 | 1437 | 7 | 285 | 7 |
| 62 | 5 | 3 | 1 | 3 | 28865 | 17124 | 11553 | 2614 | 1037 | 6 | 104 | 5.73 |
| 63 | 5 | 1 | 1 | 3 | 5627 | 411 | 3112 | 973 | 198 | 8 | 54 | 19.97 |
| 64 | 5 | 2 | 2 | 3 | 3200 | 838 | 1818 | 58 | 71 | 6 | 60 | 5.47 |
| 65 | 5 | 3 | 1 | 3 | 213924 | 142045 | 5493 | -2120 | 272 | 6 | 126 | 31 |
| 66 | 5 | 9 | 1 | 3 | 80913 | 37219 | 54462 | 8615 | 3475 | 6 | 390 | 5.55 |
| 67 | 5 | 6 | 1 | 3 | 15683 | 8968 | 18593 | 2618 | 63 | 6 | 164 | 12 |
| 68 | 5 | 5 | 1 | 3 | 18565 | 7796 | 37026 | 1395 | 798 | 6 | 144 | 3.2 |
| 69 | 5 | 1 | 1 | 3 | 3651 | 1398 | 2477 | 126 | 4 | 13 | 31 | 10.47 |
| 70 | 5 | 7 | 1 | 3 | 17482 | 15066 | 9130 | -4079 | 85 | 6 | 150 | 7 |
| 71 | 5 | 3 | 1 | 2 | 7645 | 3801 | 4410 | 190.9 | 38 | 34 | 100 | 14.91 |
| 72 | 5 | 1 | 1 | 2 | 2226 | 1255 | 2715 | 122 | 97 | 32 | 26 | 5.4 |
| 73 | 5 | 4 | 2 | 3 | 3692 | 451 | 3372 | 486 | 337 | 4 | 110 | 6.32 |
| 74 | 5 | 1 | 1 | 3 | 2564 | 903 | 2175 | 56 | 86 | 11 | 67 | 6.4 |
| 75 | 5 | 1 | 1 | 3 | 5526 | 1066 | 6323 | 2626 | 1472 | 15 | 70 | 10.36 |
| 76 | 5 | 2 | 1 | 2 | 18365 | 5206 | 11564 | 261 | 328 | 32 | 81 | 5.32 |
| 77 | 5 | 4 | 1 | 3 | 7807 | 4124 | 3551 | 279 | 307 | 8 | 152 | 10.04 |
| 78 | 5 | 9 | 1 | 2 | 71541 | 38477 | 79202 | 5302 | 1157 | 32 | 306 | 5.6 |
| 79 | 5 | 6 | 1 | 3 | 24774 | 15053 | 18523 | 2123 | 408 | 6 | 171 | 4.43 |
| 80 | 5 | 6 | 1 | 2 | 22704 | 1386 | 19694 | 5115 | 779 | 32 | 192 | 4.55 |
| 81 | 5 | 1 | 1 | 2 | 1389 | 775 | 4281 | 141 | 115 | 34 | 26 | 5.9 |
| 82 | 5 | 1 | 2 | 3 | 4513 | 3854 | 2000 | 139 | 37 | 6 | 34 | 9 |
| 83 | 5 | 1 | 1 | 2 | 8273 | 4812 | 5000 | 510 | 183 | 32 | 65 | 10.88 |
| 84 | 6 | 3 | 1 | 2 | 22249 | 5289 | 12421 | 1503 | 807 | 32 | 115 | 4.24 |
| 85 | 6 | 2 | 2 | 3 | 4665 | 1610 | 1211 | 2 | 16 | 6 | 98 | 8.6 |
| 86 | 6 | 4 | 1 | 3 | 4686 | 2418 | 35236 | 1103 | 141 | 16 | 121 | 3.24 |
| 87 | 7 | 5 | 1 | 2 | 5420 | 3789 | 5175 | 94 | 543.01 | 34 | 126 | 7.01 |
| 88 | 7 | 3 | 1 | 3 | 10056 | 4543 | 22227 | 115 | 69 | 6 | 122 | 3.3 |
| 89 | 7 | 6 | 1 | 3 | 39387 | 8157 | 11124 | 4088 | 926 | 6 | 180 | 6.12 |
| 90 | 7 | 4 | 2 | 3 | 24642 | 12447 | 7451 | 198 | 468 | 6 | 184 | 9.7 |
| 91 | 7 | 2 | 1 | 3 | 16636 | 3119 | 9786 | 1817 | 714 | 6 | 79 | 6 |
| 92 | 7 | 6 | 1 | 3 | 16966 | 2166 | 61360 | 1240 | 138 | 6 | 212 | 5.16 |
| 93 | 7 | 5 | 1 | 3 | 11348 | 2246 | 6397 | -780 | 885 | 6 | 145 | 38.04 |
| 94 | 7 | 4 | 1 | 3 | 31483 | 24696 | 16015 | 1435 | 1472 | 6 | 123 | 4.95 |
| 95 | 8 | 5 | 1 | 2 | 23099 | 12106 | 15042 | 686 | 417 | 32 | 157 | 5.12 |
| 96 | 8 | 4 | 1 | 3 | 68026 | 27724 | 56139 | 2368 | 1131 | 6 | 142 | 3.83 |
| 97 | 8 | 1 | 2 | 3 | 1529 | 673 | 1493 | 41 | 129 | 4 | 16 | 8 |
| 98 | 8 | 2 | 1 | 3 | 8002 | 2975 | 8970 | 832 | 44 | 14 | 69 | 6.1 |
| 99 | 8 | 8 | 1 | 3 | 36111 | 10307 | 22391 | 9106 | 2579 | 6 | 287 | 6.81 |
| 100 | 8 | 1 | 2 | 3 | 1948 | 1051 | 2348 | 269 | 60 | 3 | 16 | 5.97 |
| 101 | 8 | 1 | 1 | 3 | 3442 | 1911 | 2028 | 495 | 297 | 1 | 46 | 12.6 |
| 102 | 8 | 3 | 1 | 3 | 10700 | 4929 | 6482 | 225 | 361 | 6 | 96 | 7.3 |
| 103 | 8 | 2 | 1 | 3 | 13896 | 5603 | 17344 | 1873 | 1080 | 9 | 104 | 7.1 |
| 104 | 8 | 2 | 1 | 2 | 9179 | 5900 | 3099 | 118 | 82 | 32 | 89 | 20.8 |
| 105 | 8 | 5 | 1 | 3 | 22076 | 1203 | 19690 | 2663 | 822 | 6 | 127 | 4.03 |
| 106 | 8 | 3 | 1 | 2 | 29364 | 5831 | 15388 | 3370 | 367 | 31 | 118 | 8.87 |
| 107 | 8 | 1 | 1 | 3 | 2395 | 1462 | 2015 | 171 | 69 | 10 | 34 | 8 |
| 108 | 8 | 1 | 1 | 2 | 7161 | 2288 | 3404 | 270 | 297 | 33 | 70 | 8.6 |
| 109 | 8 | 2 | 1 | 2 | 1890 | 726 | 2558 | 105 | 33 | 34 | 76 | 6.5 |
| 110 | 8 |  | 1 | 2 | 26315 | 9164 | 11505 | 1009 | 288 | 32 | 471 | 3 |
| 111 | 8 | 5 | 1 | 3 | 10265 | 5113 | 11523 | 217 | 173 | 6 | 159 | 4.5 |
| 112 | 8 | 1 | 1 | 3 | 5627 | 3628 | 1661 | -298 | 96 | 12 | 48 | 23.36 |
| 113 | 8 | 1 | 1 | 3 | 7452 | 3911 | 5085 | 986 | 183 | 7 | 75 | 6.89 |
| 114 | 8 | 3 | 1 | 2 | 8503 | 391 | 7698 | 684 | 283 | 32 | 98 | 5.04 |
| 115 | 8 | 18 | 1 | 3 | 176930 | 32207 | 70895 | 18748 | 11396 | 7 | 1023 | 15.84 |
| 116 | 8 | 11 | 1 | 3 | 31161 | 15091 | 20712 | 4846 | 1066 | 9 | 380 | 9.06 |
| 117 | 8 | 1 | 1 | 3 | 4197 | 1975 | 2342 | 64 | 36 | 10 | 47 | 9 |
| 118 | 8 | 1 | 1 | 3 | 2336 | 443 | 2189 | 280 | 135 | 6 | 33 | 7 |
| 119 | 8 | 3 | 2 | 3 | 11421 | 6506 | 1971 | 10 | 12 | 4 | 76 | 8.73 |
| 120 | 8 | 16 | 1 | 3 | 64205 | 37279 | 65412 | 7401 | 2899 | 6 | 581 | 4.33 |
| 121 | 8 | 2 | 1 | 3 | 10963 | 5147 | 2619 | -741 | 240 | 6 | 78 | 8.53 |
| 122 | 8 | 3 | 1 | 3 | 10565 | 2219 | 5968 | 1827 | 747 | 19 | 96 | 6.26 |
| 123 | 8 | 1 | 1 | 2 | 1398 | 621 | 4026 | 93 | 53 | 29 | 33 | 7 |
| 124 | 8 | 1 | 2 | 3 | 3609 | 3869 | 930 | -463 | 47 | 2 | 42 | 15 |
| 125 | 8 | 7 | 1 | 2 | 31091 | 3098 | 7799 | 273 | 823 | 33 | 206 | 14.6 |
| 126 | 8 | 3 | 1 | 3 | 15408 | 10270 | 14457 | 1989 | 864 | 6 | 111 | 7 |
| 127 | 8 | 1 | 2 | 3 | 762 | 33 | 1429 | -16 | 20 | 4 | 30 | 5.7 |
| 128 | 8 | 4 | 1 | 3 | 11706 | 4411 | 7014 | 388 | 158 | 10 | 142 | 5 |
| 129 | 8 | 16 | 1 | 3 | 57407 | 23832 | 48955 | 6127 | 2254 | 6 | 588 | 3.83 |
| 130 | 8 | 2 | 1 | 3 | 13881 | 6506 | 4338 | 200 | 139 | 21 | 84 | 5.2 |
| 131 | 8 | 1 | 2 | 3 | 2613 | 1406 | 2083 | 22 | 1 | 4 | 15 | 6.7 |
| 132 | 8 | 1 | 1 | 3 | 22426 | 16582 | 27414 | 988 | 757 | 11 | 36 | 4.09 |
| 133 | 8 | 1 | 2 | 3 | 16285 | 7765 | 12533 | 4131 | 594 | 21 | 61 | 4.09 |
| 134 | 8 | 1 | 1 | 3 | 23150 | 522 | 21432 | 629 | 531 | 17 | 68 | 4.02 |
| 135 | 8 | 13 | 1 | 2 | 170580 | 25650 | 17632 | 1382 | 2836 | 30 | 389 | 6.7 |
| 136 | 8 | 2 | 1 | 2 | 8424 | 1666 | 11289 | 3897 | 1339 | 30 | 70 | 4.05 |
| 137 | 8 | 1 | 1 | 3 | 6295 | 137 | 2018 | 972 | 363 | 1 | 12 | 14.7 |
| 138 | 8 | 1 | 1 | 2 | 2879 | 1444 | 2742 | 99 | 83 | 32 | 28 | 7.24 |
| 139 | 8 | 3 | 1 | 3 | 14058 | 6833 | 6455 | 105 | 448 | 21 | 114 | 7.9 |
| 140 | 8 | 11 | 1 | 3 | 77873 | 36001 | 21824 | 1507 | 1757 | 6 | 383 | 25.26 |
| 141 | 8 | 7 | 1 | 3 | 15848 | 5718 | 10111 | -842 | 920 | 7 | 254 | 6.7 |
| 142 | 9 | 4 | 1 | 3 | 18640 | 5492 | 12459 | 1259 | 628 | 6 | 150 | 7 |
| 143 | 9 | 23 | 2 | 3 | 40065 | 14428 | 30349 | 3841 | 2608 | 4 | 1404 | 7.48 |
| 144 | 9 | 1 | 1 | 3 | 5648 | 1357 | 4707 | 1054 | 361 | 21 | 43 | 7.22 |
| 145 | 9 | 2 | 1 | 3 | 10310 | 2065 | 9818 | 83 | 90 | 6 | 58 | 6.08 |
| 146 | 9 | 3 | 1 | 2 | 28995 | 5886 | 32926 | 6567 | 1763 | 32 | 150 | 5 |
| 147 | 9 | 3 | 1 | 3 | 6307 | 2459 | 2113 | 27 | 5 | 8 | 115 | 9 |
| 148 | 9 | 1 | 1 | 3 | 11678 | 1789 | 10352 | 1334 | 471 | 6 | 40 | 4.8 |
| 149 | 9 | 1 | 1 | 2 | 9975 | 4170 | 5988 | 42 | 217 | 32 | 69 | 6.6 |
| 150 | 9 | 3 | 1 | 3 | 17061 | 10989 | 2752 | -4134 | 0 | 1 | 106 | 14.25 |
| 151 | 9 | 11 | 1 | 3 | 23683 | 5322 | 14444 | 10474 | 6472 | 21 | 301 | 4.85 |
| 152 | 9 | 3 | 1 | 3 | 20868 | 6506 | 8641 | 1885 | 741 | 6 | 94 | 6.07 |
| 153 | 9 | 1 | 1 | 3 | 6932 | 798 | 15348 | 4066 | 651 | 24 | 89 | 4.53 |
| Total | Average value | 13.013 | 1.144 | 2.791 | 23808.618 | 9760.971 | 14845.505 | 1761.808 | 746.882 | 155.373 | 8.401 | 13.013 |
|  | Standard deviation | 10.500 | 0.352 | 0.424 | 37433.022 | 17304.930 | 20756.209 | 3669.338 | 1266.081 | 201.689 | 6.568 | 10.500 |
|  | Variance | 110.250 | 0.124 | 0.180 | 1401231114.965 | 299460589.433 | 430820210.588 | 13464038.330 | 1602961.065 | 40678.485 | 43.144 | 110.250 |

**Note:** The number of Dadong district, Heping district, Huanggu district, Hunnan district, Shenbei new district, Shenhe district and Sujiatun district, Tiexi district, and Yuhong district are 1, 2, 3, 4, 5, 6, 7, 8, 9, respectively.
